# Importance of Plants with Extremely Small Populations (PSESPs) in Endemic-Rich Areas, Elements Often Forgotten in Conservation Strategies

**DOI:** 10.3390/plants10081504

**Published:** 2021-07-22

**Authors:** Donatella Cogoni, Giuseppe Fenu, Carlo Dessì, Angela Deidda, Cesario Giotta, Marcello Piccitto, Gianluigi Bacchetta

**Affiliations:** 1 Centro Conservazione Biodiversità (CCB), Dipartimento di Scienze della Vita e dell’Ambiente, Università degli Studi di Cagliari, Viale Sant’Ignazio da Laconi 13, 09123 Cagliari, Italy; d.cogoni@unica.it (D.C.); bacchet@unica.it (G.B.); 2 Dipartimento di Scienze della Vita e dell’Ambiente, Università degli Studi di Cagliari, Viale Sant’Ignazio da Laconi 13, 09123 Cagliari, Italy; 3 Independent Researcher, Vico Sant’ Ignazio 22, 09090 Laconi, Italy; carlettodessi60@gmail.com; 4 Independent Researcher, Via Frumini 13, 09090 Laconi, Italy; angeladei@tiscali.it; 5 Independent Researcher, Viale Marconi 82, 08045 Lanusei, Italy; cesariogiotta@tiscali.it; 6 Istituto d’Istruzione Superiore Leonardo da Vinci, Via Leonardo da Vinci1, 08045 Lanusei, Italy; marcellopiccitto@tiscali.it

**Keywords:** evolutionary potential loss, knowledge gaps, Mediterranean flora, monitoring activities, *Ophioglossum vulgatum* L., plant conservation, Sardinia

## Abstract

The distribution of the threatened fern *Ophioglossum vulgatum* L., a plant with extremely small populations (PSESPs) in Sardinia, is characterized by small disjunct populations with only a few individuals, and little is known about its status in the wild. To provide information for the conservation of *O. vulgatum* and with the aim to develop an in situ conservation strategy, we investigated its distribution, population size, and habitat. Field surveys confirmed that the species grows in only five localities. Two representative populations were selected for this study (Funtanamela and Gedili), and in each population, all plants were mapped and monitored monthly from April to August over an 8-year period. During the study, the populations had a very low number of reproductive plants and the populations appeared to be in decline, with the total number of plants per population slightly decreased in Gedili while a sharp reduction was recorded in Funtanamela due to wild boar threat. A fence was built in order to protect the site from further damage, but no noticeable signals of recovery were observed. The most urgent conservation requirement for this species is to preserve the threatened habitat of the remnant populations. Further field surveys and research are also required for an improved understanding of the species’ status.

## 1. Introduction

Island ecosystems have always played a leading role in conservation biology, and generally are hotspots of biodiversity [1,2,3], with legacies of relatively recent human impact and native species’ extinctions, and therefore provide significant challenges when considering how to conserve biodiversity. They also offer some of the best-suited scenarios for rapidly advancing our understanding of fundamental aspects of human relationships with nature, and of conservation strategies [4].

Despite the high biodiversity value, island plant diversity is severely threatened both by natural and anthropogenic factors, including geological events, plant-animal interactions, stochastic events, species invasions, land-use change, habitat destruction or fragmentation, overharvesting for economic purposes, and global climate change (e.g., [5,6,7,8,9,10,11]). Indeed, it is widely recognized that most Earth ecosystem processes are being altered by human activities, suggesting that we may have entered a human-dominated geological epoch called the “Anthropocene” [12], and that humans are causing the sixth mass species extinction (e.g., [13,14]). These serious threats are the cause of numerous extinctions recognized in insular context worldwide [15,16,17,18], and their knowledge can be considered a clarion call to increase global efforts to study, halt, and possibly reverse the ongoing negative environmental trends [14].

Mediterranean islands encompass a wide range of habitats within a small and restricted range; past geographical and climatic changes, combined with current environmental heterogeneities, have molded, within them, unusually high levels of biodiversity, which is very important for their own value of biodiversity, both in term of plant species (i.e., numerous endemics, presence of “climate relicts”) and of ecosystems’ assemblage [2,6,19,20]. As they comprise a variety of valuable habitats, insular ecosystems are considered more fragile than continental ones, while the uncertainty regarding the conservation of valuable native flora is much more exacerbated in insular habitats than in their mainland counterparts [21].

Sardinia, the second largest island in the Mediterranean Basin, is exceptionally rich in plant diversity, with more than 2450 vascular taxa [22]; among them, 295 are endemics (189 exclusive Sardinian endemics, 90 Sardinian–Corsican endemics, and 16 that are also present in the Tuscan Archipelago) as a result of the geographical isolation and a high geological and geomorphological diversity that have contributed to the formation of a wide range of habitats and consequent high rate of endemism (e.g., [3,23]). In such endemic-rich areas, the conservation concerns are so far all focused on the endemic component, since these species are usually better studied and frequently more threatened than the non-endemic flora and constitute a central group for conservation [3,21], since the conservation of biodiversity occurs via the implementation of policy with only limited resources [24,25,26]. In this context, Sardinia is not an exception: despite the high level of uniqueness in the vascular flora, until a few years ago not many conservation studies and actions had been carried out on threatened plant species, and the few studies were mainly oriented on extremely narrow endemic and endemic plants. Only in the last years has a trend inversion been registered, with an increasing number of papers and conservation actions detected about the threatened flora, indicating that greater attention is being paid to threatened plants and, in general, to the island’s plant diversity (e.g., [21,27]). Nonetheless, mainly due to limited human and economic resources (e.g., [24,25]), these conservation achievements are not enough to protect Sardinia’s high level of plant diversity, therefore conserving the most threatened wild species in the island is more urgently needed than ever before.

Often, in addition to the main human-related threats, the progress of plant conservation action is also hindered by several additional factors, such as management issues (e.g., lack of conservation awareness from governmental officials and local people), which create barriers to conservation success, because very often the importance of conserving even a single species is ignored if its value is not known [28]. It is therefore essential to create a “priority list” of plants for which protection is needed through “regional responsibility” criteria to identify the target species which enrich the levels of biodiversity of a territory [21,29,30,31,32]. Unfortunately, as in several Mediterranean areas, in Sardinia there are regional gaps in related laws and regulations regarding wild plant conservation. Furthermore, these lists must be open and include not only endemic species but also other plants of conservation interest, as suggested for the Mediterranean insular floras [21], and, when possible, be capable of highlighting the conservation needs by single population rather than by species [33].

As current conservation efforts of governments, scientists, conservationists, and the general public are often focused on endemic, threatened, and endangered plants, one of the main difficulties is understanding, beyond these species, which wild species should receive protection priority as they favor the values of biodiversity. Among these, wild plants with extremely small populations (PSESPs) deserve conservation interest and have a critical role for sustainable development and for saving biodiversity [28,34,35]. To focus on plants with extremely small populations with in situ and ex situ conservation action is another fundamental pillar to preserve the level of plant diversity of a region [28,34,35]. The PSESP concept refers to species with low numbers and with much lower than minimum viable populations for long-term survival in the wild, due to serious human disturbance in recent times; it excludes naturally rare species. Small remaining populations, restricted habitat, serious human disturbance, and extremely high risk of extinction are key characteristics of PSESPs [28]. Therefore, it is important to study and conserve plant species with small populations, although their distributions are over large territories, not only for their contribution to increase the floristic richness of a territory, but especially for their evolutionary potential [19].

An iconic case of such type of plants is represented by the small fern *Ophioglossum vulgatum* L. (Ophioglossaceae), widely distributed in the temperate regions of the Northern Hemisphere but present only in a few disjunct localities in Sardinia, always in extremely small populations in size. For this fern that has a wide distribution, conservation has not been a priority so far, and very little is known about its ecology and distribution, as well as the population size and dynamic in Sardinia, and even less about its conservation status. In addition to the lack of this information, for Sardinian populations the problems related to its conservation are both the lack of legal regulation that dictate the need for conserving species, and protocols/methodologies for conserving peculiar species that are inadequate and usually do not specify how conservation should take place. In the frame of conservation activities carried out in Sardinia for the endangered flora, and considering the lack of ecological and distributive studies, an extensive monitoring plan for this species started in 2011 with the main aim to develop an in situ conservation strategy. Specifically, this study aimed to provide detailed information about the current status of two selected small populations of this wild plant in Sardinia, subjected to several threats and, consequently, whose conservation is a priority at the regional level. Further aims were to analyze the need for its protection and recommend appropriate conservation measures, therefore we estimated the species’ distribution and the single population size, characterized its habitat, and monitored all populations over time.

## 2. Results

**Figure 1:**
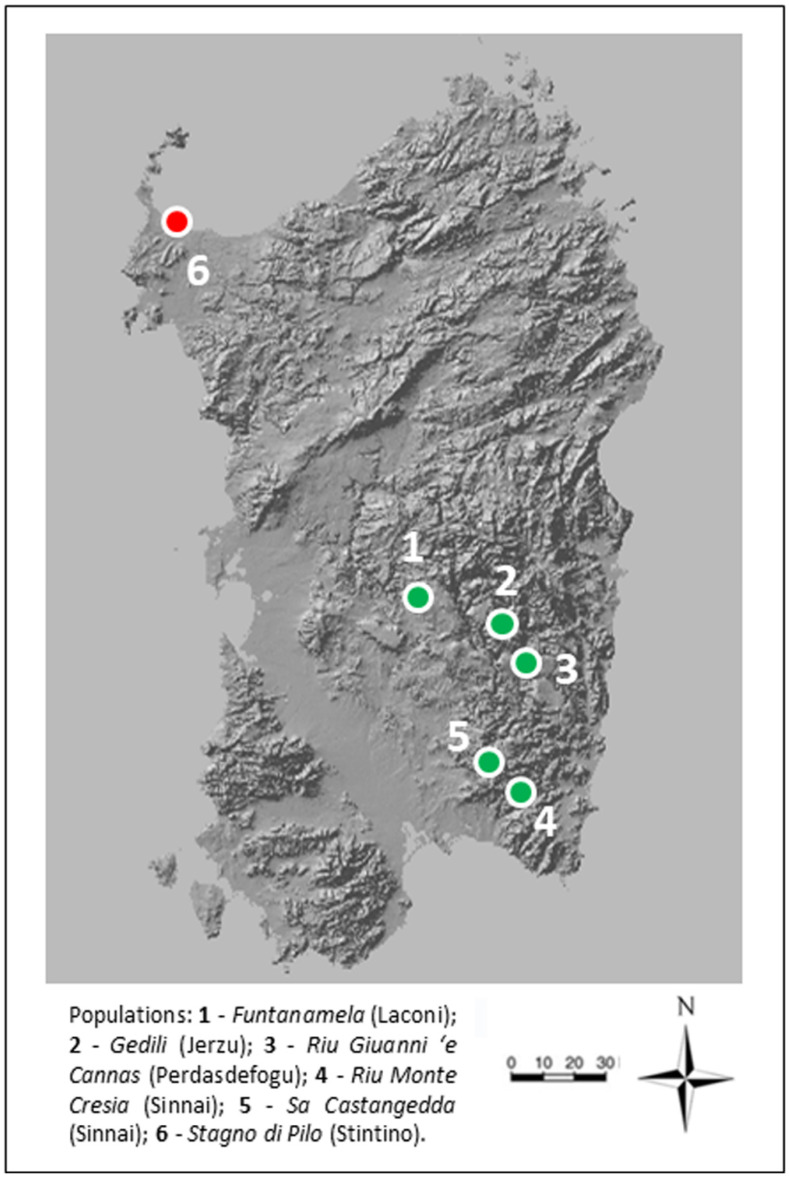
Current distribution of *Ophioglossum vulgatum* L. in Sardinia: confirmed populations are in green and (probably) extirpated in red.

As a result of our study, and considering both historical and newly discovered populations, *O. vulgatum* was recorded (and confirmed) in five Sardinian localities; in particular, three of them reported in bibliography for Funtanamela [36], Riu Monte Cresia and Sa Castangedda [37], and two for which old herbarium specimens had been preserved in *Herbarium* FI (Gedili; Piccitto and Giotta, 28.VI.1995) or CAG (Riu Giuanni ‘e Cannas; Lai, 15.V.1998; Table 1 and Figure 1). An old herbarium specimen of *O. vulgatum*, collected in 1967 and stored at the *Herbarium* CAG, comes from a coastal brackish pond located in the northwest of the island, near Stintino (Table 1).

In Sardinia, *Ophioglossum vulgatum* currently grows on various lithological substrata (carbonate and intrusive), where a pedogenetically evolved humus-rich soil horizon was present, in flat areas at altitudes ranging from 630 to 760 m a.s.l. (Table 1). In the island, this species usually occurs in humid meadows habitats, often under riparian formations characterized by peculiar species such as *Taxus baccata* L. and *Cornus sanguinea* L. All populations of *Ophioglossum vulgatum* grow in areas that can be referred to as the Mediterranean euoceanic bioclimate and, more specifically, a lower meso-Mediterranean thermotype and lower subhumid ombrotype.

All populations of this rare fern are characterized by a low number of reproductive plants which, on average, varies between 25 and 150 individuals, depending on the years. No population contained >250 mature individuals, with a general decreasing trend over the study period (Table 1).

**Figure 2:**
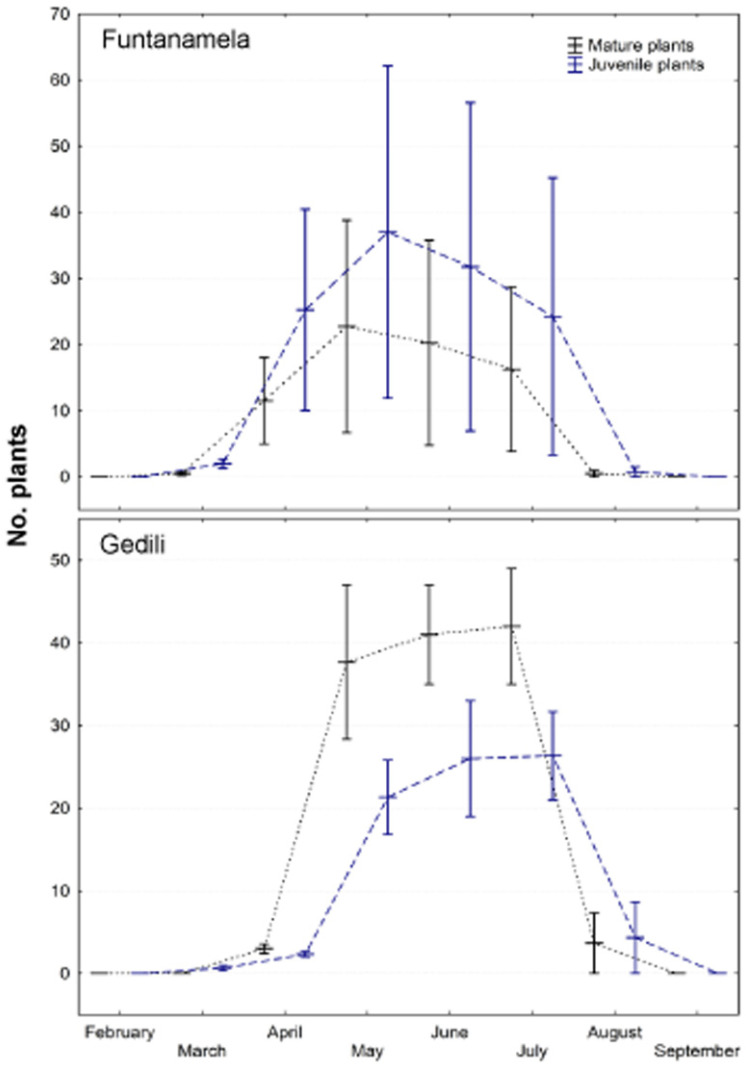
Mean values (±SE) of juvenile and mature plants per month in Funtanamela (Laconi) and Gedili (Jerzu) during the 8-year period of monitoring.

The detailed monitoring showed that the vegetative season started in early March for both populations, but the maximum number of individuals per population was observed in May (Funtanamela) or July (Gedili); the population structure showed a dominance of juvenile plants in Funtanamela and mature individuals in Gedili (Figure 2).

**Figure 3:**
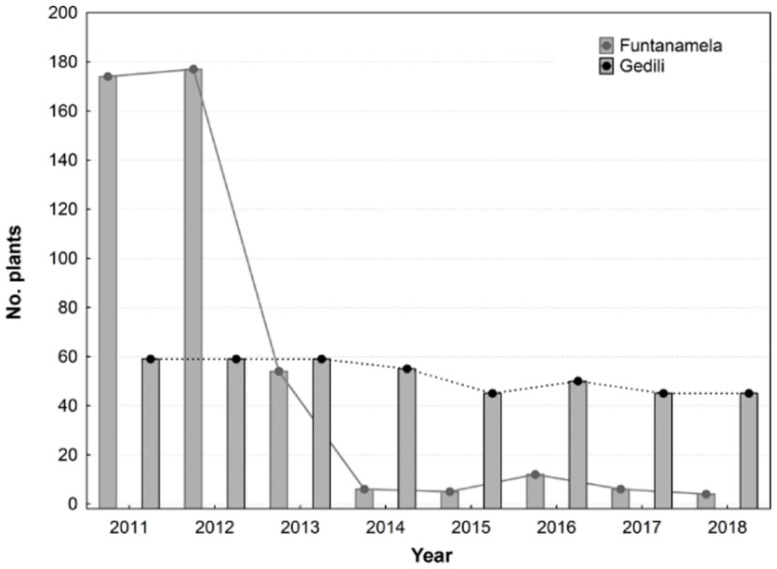
Population size per year in Funtanamela (Laconi) and Gedili (Jerzu) during the 8-year period of monitoring.

**Figure 4:**
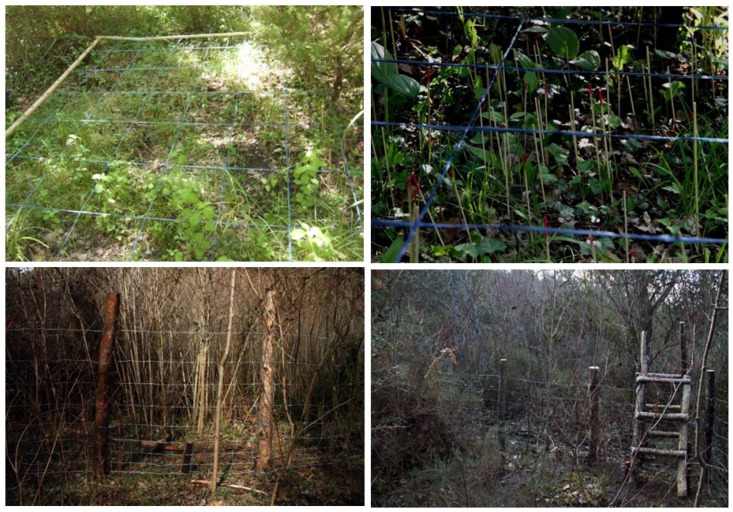
Monitoring protocol using a fixed grid to map each individual plant and different woody stock to distinguish juvenile and mature plants (up); fence protection for the population of *Ophioglossum vulgatum* at Funtanamela, Laconi (Photo by Giuseppe Fenu and Carlo Dessì).

In 2011, the initial number of monitored individuals varied from 177 to 49 plants in Funtanamela and Gedili, respectively. In winter 2013, before the growing season began, the site where *O. vulgatum* population grow in Funtanamela had been severely damaged by the rooting activity of wild boars, with a consequent drastic reduction in population size in the following spring (Figure 3). Despite this situation, the University of Cagliari continued the periodic monitoring of both populations. In 2016, in the frame of the Care-Mediflora project (http://www.CARE-MEDIFLORA.eu/, accessed on 9 June 2021), a fence was built in order to protect the site from further damage by wild boars and to observe whether the *O. vulgatum* population was able to recover itself naturally (Figure 3 and Figure 4). During the study period, the total number of plants per population slightly decreased in Gedili, while a sharp reduction was recorded in Funtanamela following the event mentioned above. As a consequence of the damage, in this population, <10 individuals were always observed both before and after the fence erection; in addition, the number of reproductive plants never exceeded two units, being zero in 2014, 2016, and 2017. During the last monitoring in Funtanamela (2019), six years after the population extirpation and four years after the fence construction, no noticeable signals of recovery were observed.

## 3. Discussion

Key characteristics of PSESPs are the small remaining populations, often restricted habitat, serious natural and/or human disturbance, and extremely high risk of extinction [28]; therefore, it is important to study and conserve plant species with a small population, although their distribution is over large territories, not only for of their contribution to increase the floristic richness of a territory, but especially for their evolutionary potential [19]. *O. vulgatum* in Sardinia showed all the typical traits of a PSESP in terms of small and isolated populations, peculiar ecological requirements, high rate of natural/human disturbance, and a probable case of local extirpation associated with a lack of attention to conservation. Our long-term study allowed us, first of all, to obtain a current distribution of this species on the island and detailed ecological and population data. It was interesting to observe how the population structures were different in the two monitored sites with a typical structure of an expanding population in Funtanamela (high number of juvenile plants), against a more stable population in Gedili (high number of mature plants). Although further analyses are necessary, these differences could be due to local ecological situations that become decisive in determining population dynamics, as often happens for small populations (e.g., [38,39]). A separate discussion must be had for the population in the Stagno di Pilo, for which there are no recent confirmations: the herbarium specimen is correctly determined, and the collector was certainly an expert botanist; in addition, it has also been demonstrated that *O. vulgatum* can grow on sandy habitats and tolerate increased NaCl content in the soil. For these reasons, it is conceivable that this population is extinct due to anthropogenic activities or to some stochastic event such as that observed in Funtanamela.

Some clues for conservationists can be obtained from our long-term monitoring activity. First of all, natural stochastic events can lead to a strong reduction of a plant population or a local extinction; this is particularly relevant when these events occur to the detriment of plants with extremely small populations, such as *O. vulgatum* in Sardinia. These events, often negligible in space and time, can therefore lead to a loss of biological diversity in a territory, often without the awareness on the part of conservationists. In this context, a peculiar role is linked to the high abundance of ungulates [40,41,42,43]; the wild boar (*Sus scrofa*) activity that affects several taxa both directly and indirectly (e.g., through predation and/or alteration of different ecosystem parameters) is the rooting activity, as this may alter soil and vegetation and overturn extensive areas [40,41,42]. In fact, this species is considered one of the most invasive species in terms of impact on biological diversity, especially because the population size has increased rapidly in recent decades in several regions of the world [41,42,43,44]. To our knowledge, the effects of rooting activity on narrow endemic plants and/or plants with extremely small populations are poorly documented in literature; in our study case, the rooting activity seems to result in a local extirpation of a small population, since no significant recovery of the population has been observed, despite the protections.

Practical conservation actions, such as protective fence erection, may be useless in saving a compromised situation, or can take many years to show their effectiveness. The implementation of passive protection measures represents an emergency solution to limit overgrazing, but comprehensive measures, such as herbivore control (both wild and domestic) accompanied by species-specific translocation, are always preferable [45]. More in general, the use of protective fences to protect threatened populations is a topic currently highly debated among conservationists, and there is no unanimous consensus on the real effectiveness of these measures [45]. As mentioned above, actions can take many years to show their effectiveness. In this specific case, it takes a long time and a lot of constancy in monitoring activities to understand whether the passive defense measures undertaken can be successful on a small population. Therefore, conservation programs should be designed with viable, long-term measures in mind. In addition to the long-term monitoring, at the same time, the evaluation methods for enhancing protection effectiveness are essential to ensure the effectiveness of the conservation measures.

The few remaining populations of *O. vulgatum* require urgent conservation action, both to protect and reinforce extant populations and, if possible, to reintroduce new populations into suitable sites. To do this, although there are no specific protocols, the experiences gained in recent years on other small ferns could be exploited (e.g., [46,47]).

A further indication is that populations growing on land managed by public administrations are not always more protected, per se, than those growing on private land; it is unanimously accepted that carrying out in situ conservation actions on public land is easier (e.g., [45,48]), but it is now necessary to also address the issue with the owners of the land where threatened plant populations grow: this is certainly a great challenge for the future that can only be won by involving people and implementing awareness on the challenge of biodiversity conservation.

Unfortunately, although PSESPs have received much attention, especially in China [28,34,49,50,51,52], conservation of endangered plants with extremely small populations is particularly difficult, especially because there are few successful examples to follow (e.g., [44,45]), and because most wild plant species with extremely small populations are not legally included in official protection programs [34]. In addition, so far no detailed and appropriate protocols/methodologies for conserving PSESPs are available in literature [53]. All these aspects considered, the establishment of small-scale reserves or plant micro-reserves must be recognized as the most appropriate precautionary approach for in situ conservation of PSESPs; against this background, continuous long-term monitoring is essential for successful in situ conservation management of PSESPs (e.g., [53,54]).

Our study demonstrates that the conservation status of a plant globally considered not threatened could notably vary at a small spatial scale; accordingly, such evidence leads us to confirm that conservation priorities should vary at the small local level, since a population could need different conservation measures depending on the particular local conditions [21,33]. Because these species are generally not included in specific regional conservation strategies, most plant species with extremely small populations remain in danger of extinction; a first step at the local level in this direction is the inclusion of these plant species in the regional priority lists (according to the “regional responsibility” criterion) and in the National Red Lists.

In the inexorable homogenization of global biodiversity, plant diversity loss is manifested most obviously and most consistently by the disappearance of rare species such as PSEPs; against this background, conservation action must have a focus on the long-term future of these species to save them wherever and whenever possible in the wild [54]. Ensuring a secure long-term future for plants such as *O. vulgatum* in Sardinia has important intrinsic value since they encapsulate millions of years of evolutionary history.

## 4. Materials and Methods

**Figure 5:**
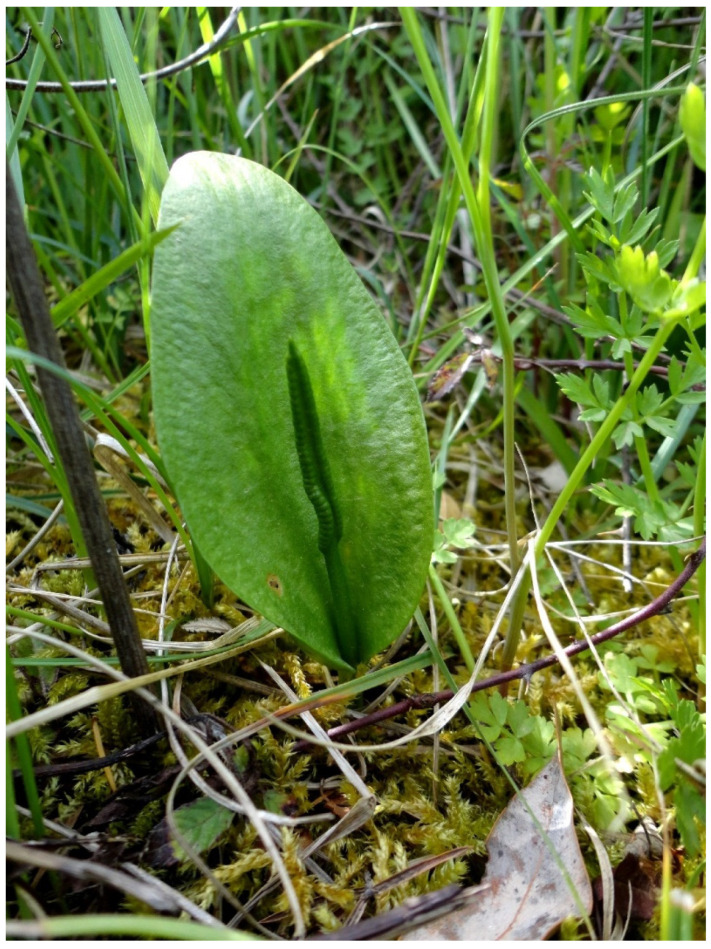
*Ophioglossum vulgatum* L. detail (photo by Giuseppe Fenu).

*Ophioglossum vulgatum* L. (Figure 5) is a terrestrial, homosporous, perennial geophyte, 5–30 cm tall, with a single sterile leaf. The spike consists of a variable number, between 10 and 40, of segments on each side [55,56]. The rhizome is usually short and erect, bearing one, rarely two, fronds. The frond is simple, entire, and ovate, 2–5 × 3–12 cm, with an adaxially placed fertile spike, showing 15–50 sporangia on each side [55,56]. *Ophioglossum vulgatum* is a small fern, the sporophyll sprouts out in April–June and wind is its main spore dispersal agent. This cosmopolitan plant species occurs throughout its range in such diverse habitats as fens, damp sands, pastures, wet meadows, grassy swales, moist woods, rich swamplands, and mud creeks; occasionally it occurs on rocky hillsides or on dry, sandy beaches where it tolerates increased NaCl content in the soil (e.g., [56,57]). In Europe, *Ophioglossum vulgatum* usually grows in a variety of wet and mesic habitats, including wet and peaty meadows, marshes, stream edges, and hygrophilous woodlands from sea level up to 1800 m [56,57]; it is generally considered as a characteristic species of the wet and oligotrophic *Molinion caeruleae* alliance [57], which, according to the 92/43/EEC Directive, refers to the habitat of European interest “Molinia meadows on calcareous, peaty or clayey-silt-laden soils (*Molinion caeruleae)*” (code 6410). Mainly due to its wide distribution, *O. vulgatum* has been assessed as LC at European level [58], nevertheless, currently ferns and lycophytes have to face a new kind of risk related to ongoing climate change and human-related environmental disturbances such as eutrophication, pollution, habitat loss, alteration of hydrological regimes, and overexploitation [59,60,61,62]. Few data were available on the distribution of this small fern, and the conservation status at regional level (Italian and/or Sardinian) was not yet assessed.

Data on the geographical distribution, ecology, and status of *O. vulgatum* populations in Sardinia were collected by both bibliographic, herbarium specimens or database records and fieldwork carried out during the last ten years. Specifically, field surveys were focused on the localities for which herbarium specimens and other bibliographic data were available, and in other areas ecologically suitable where this species could potentially occur. When a locality was confirmed or discovered, the following analyses were undertaken. The geographical limits of localities were mapped each year and areas visually estimated to detect any annual changes in area occupied. For each locality, we noted the altitudinal range, slope, aspect, and habitat type.

Population size was determined by a direct count of the total number of plants, distinguishing the juvenile from the mature individuals. Among all the confirmed populations, as part of the monitoring and conservation activities carried out on the Sardinian flora of conservation interest, two representative populations were selected based on ease and feasibility to be monitored over time: one on land managed by a public administration (Funtanamela), and one on a private land (Gedili). Following the same protocol developed and tested for other small plants in Sardinia [63,64], in each population, all plants were monthly monitored from April to August over an 8-year period. During each monitoring activity, all plants were counted, marked, and the reproductive status was assessed; all new seedlings that appeared inside the plots were also counted, measured, and mapped.

## Figures and Tables

**Table 1:** Current situation in term of distribution and ecology of *O. vulgatum* in Sardinia with mean altitude, slope, aspect, lithological substrata, habitat, land management, first reporting, and current status of each locality where the species grows.

Locality (Municipality)	Mean Altitude (m a.s.l.)	Substrate	Habitat	Area (m^2^)	Land Management	First Reporting	Current Status
Funtanamela (Laconi)	726	Travertine	Riparian woods	10	Public	[36]	Confirmed
Gedili (Jerzu)	760	Limestone	Wet meadows	25	Private	M. Piccitto and C. Giotta (1995)	Confirmed
Riu Giuanni ‘e Cannas (Perdasdefogu)	630	Limestone	Wet meadows	400	Public	R. Lai (2006)	Confirmed
Riu Monte Cresia (Sinnai)	660	Granite	Riparian woods	250	Public	[37]	Confirmed
Sa Castangedda (Sinnai)	690	Granite	Riparian woods	300	Public	[37]	Confirmed
Stagno di Pilo (Stintino) *	5	Alluvial deposits	Brackish pond	N/A	N/A	M. Chiappini (1967)	Not confirmed

* Herbarium specimen, collected by Chiappini (1967) and stored at *Herbarium* CAG (University of Cagliari, Italy), that has not been refound in the last 50 years.

## Data Availability

Detailed data of monitoring are available upon request.
